# The diagnostic value of iron oxide nanoparticles for imaging of myocardial inflammation – quo vadis?

**DOI:** 10.1186/s12968-015-0165-6

**Published:** 2015-07-08

**Authors:** Michael Bietenbeck, Anca Florian, Udo Sechtem, Ali Yilmaz

**Affiliations:** Department of Cardiology and Angiology, Albert-Schweitzer-Campus 1, building A1, 48149 Münster, Germany; Division of Cardiology, Robert-Bosch-Krankenhaus, Stuttgart, Germany

**Keywords:** Cardiovascular magnetic resonance, SPIO, Myocardial, Inflammation, Infarction, Monocytes

## Abstract

Cardiovascular magnetic resonance (CMR) is an integral part in the diagnostic work-up of cardiac inflammatory diseases. In this context, superparamagnetic iron oxide-based contrast agents can provide additional diagnostic information regarding the assessment of myocardial infarction and myocarditis. After intravenous administration, these nanoparticles are taken up by activated monocytes and macrophages, which predominantly accumulate in regions associated with inflammation as was successfully shown in recent preclinical studies. Furthermore, first clinical studies with a new iron oxide-complex that was clinically approved for the treatment of iron deficiency anaemia recently demonstrated a superior diagnostic value of iron oxide nanoparticles compared to gadolinium-based compounds for imaging of myocardial inflammation in patients with acute myocardial infarction. In this article, we outline the basic features of superparamagnetic iron oxide-based contrast agents and review recent studies using such nanoparticles for cardiac imaging in case of acute myocardial infarction as well as acute myocarditis. Moreover, we highlight the translational potential of these agents and possible research applications with regard to imaging and therapy.

## Introduction

### Myocardial inflammation: cause and consequences

Myocardial inflammation is associated with a broad spectrum of pathophysiological pathways and may eventually cause both severe structural and functional impairment of the heart muscle. In principle, myocardial inflammation can be triggered by various stimuli – including myocardial infections caused by pathogens such as viruses – and myocardial ischemia due to coronary artery occlusion in case of acute myocardial infarction. Such mechanisms may lead to myocardial necrosis and initiate subsequent appropriate as well as inadequate or excessive responses of the innate immune system. Adequate activation of the immune system may promote (amongst others) removal of dead cells and matrix debris. By contrast, an excessive inflammatory response may result in adverse changes of the geometry, function and structure of heart tissue, referred to as adverse remodelling [1, 2]. A detailed listing of immune effectors and regulators involved in the pathophysiological states mentioned can be found elsewhere [3, 4]. Since myocardial inflammation may affect both primarily involved necrotic areas as well as remote (seemingly) healthy tissue, subsequent myocardial remodelling may further cause hypertrophy and/or dilated chambers and/or heart failure [4–6]. Therefore, the accurate assessment of an on-going myocardial inflammation process is of paramount importance regarding appropriate diagnosis of cardiac disease as well as therapeutic decision-making and prognostic risk stratification [3, 4]. This review focuses on the diagnostic value of iron oxide nanoparticles for myocardial inflammation imaging in case of (acute) myocardial infarction and myocarditis.

### Current (conventional) assessment of myocardial inflammation

**Fig. 1:**
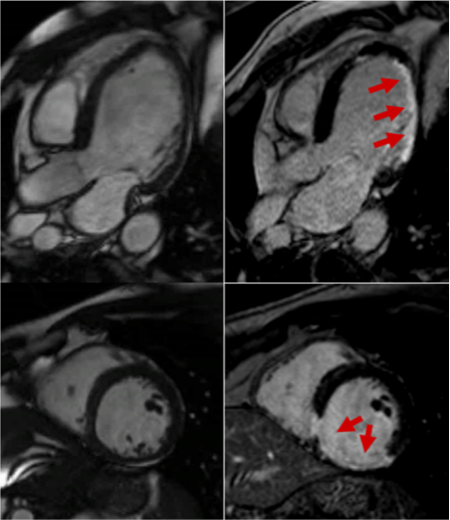
Cine-MR (left column) as well as T1-weighted inversion-recovery late gadolinium enhancement (LGE, right column) images performed in long-axis (top) and short-axis views (bottom). Damaged myocardium exhibits delayed gadolinium washout kinetics, which in turn leads to hyperintense areas (red arrows) visible in LGE images acquired 5-10 min after the injection of a gadolinium-based compound

Cardiovascular magnetic resonance (CMR) is a well-established technique in the diagnosis of cardiovascular diseases. So far, non-invasive work-up of myocardial inflammation with CMR is mainly based on observing alterations in myocardial tissue composition [7]. CMR pulse sequences applied for diagnosing myocarditis include T2-weighted imaging sequences for the unspecific diagnosis of myocardial oedema and contrast-enhanced CMR (ceCMR) for the (again unspecific) diagnosis of necrosis and/or fibrosis both of which may be present in myocardial infarction or myocarditis [5]. Gadolinium contrast media washes out rapidly in healthy myocardium whereas areas of damaged myocardium (necrosis, fibrosis or oedema) exhibit delayed washout kinetics. Thus, ceCMR may depict the area of myocardial damage 10-15 min after the injection of the contrast agent (CA) (Fig. 1) [8]. The value and accuracy of these methods and their correlation to biopsy findings in the various phases of human myocarditis are still under debate and more accurate and more specific methods for the non-invasive diagnosis of myocardial inflammation are desired [9, 10]. Particularly with regard to initiation of timely and adequate therapy, diagnosis of myocardial inflammation in the early phase of heart disease before the occurrence of structural changes in the myocardium is crucial. Unfortunately, T2-weighted pulse sequences which are theoretically best suited to depict myocardial oedema as the first morphological change in the sequence of events have been unreliable in the experience of several groups studying the diagnosis of myocarditis [11]. Hence, new CMR tools are needed to improve both early diagnosis and specificity of diagnosis. To enhance diagnostic accuracy, molecular and cellular imaging methods based on iron oxide nanoparticles (IONs) appear promising [12, 13].

### Iron oxide nanoparticles

**Fig. 2:**
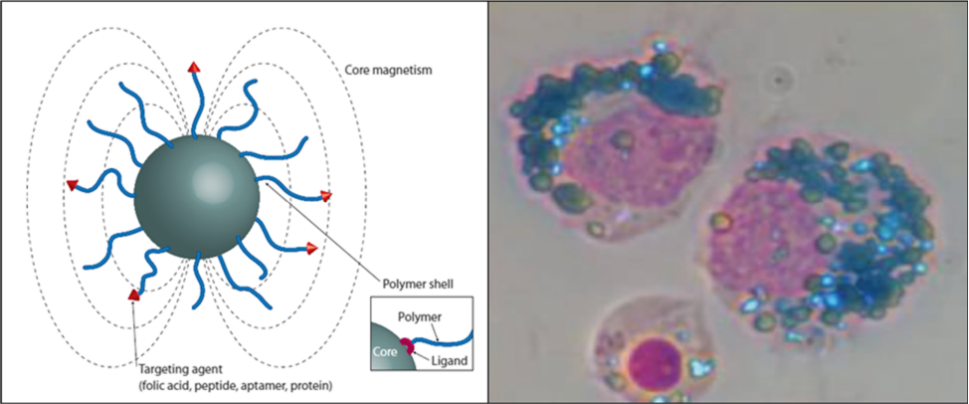
Schematic drawing of a superparamagnetic iron oxide particle (left) comprised of the magnetic core and the polymeric shell. While the core represents the active substance of the contrast agent, the coating stabilizes the particle and can be used as a grafting platform for various functional groups. Such iron oxide particles are taken up by activated monocytes (right) that can be visualized by Prussian blue staining. Reproduced with permission from Boyer et al. [61] and Richards et al. [62]

Nanoscaled particles with an iron oxide core and a polymer shell have been under investigation for the last two to three decades due to their versatile features. Probably most important, IONs exhibit superparamagnetic properties below a certain particle size. As a result, they show a much larger magnetic susceptibility than paramagnetic agents. However, they lose their magnetization in the absence of an applied magnetic field – in contrast to paramagnetic agents. An attractive feature of IONs is that, depending on their size and composition of their polymer shell, they are biocompatible and may form stable colloidal suspensions, which are equally important prerequisites for successful in vivo applications (Fig. 2). After intravenous administration, IONs disperse freely in the intravascular blood pool – until they are eventually internalized by monocytes/macrophages of the reticuloendothelial system (RES) [14]. Therefore, IONs have mainly been used for blood-pool imaging or examinations of organs associated to RES such as liver, spleen and lymph nodes. However, in the presence of an inflammatory disease, IONs are avidly taken up by activated monocytes/macrophages with subsequent accumulation in the affected organ/tissue [15]. In addition, free IONs may migrate passively across leaky endothelium at inflammatory foci and get phagocytosed by resident tissue macrophages [12]. Thus – despite remaining predominantly intravascular – IONs represent promising tracers for inflammation. Exploiting this feature and their superior magnetic properties, tracking and visualization of internalized IONs by appropriate CMR sequences have great potential in the assessment of inflammatory processes. Moreover, appropriate surface modifications can functionalize IONs for several applications besides imaging. Amongst many others, their use in targeted drug delivery, magnetofection, hyperthermia or ex vivo molecular diagnostics have been reported [16].

## Basic design of iron oxide nanoparticles

### Basic design & materials used for coating

Although IONs may differ vastly according to their desired application (see above), they share a common structure. Briefly, they are composed of a magnetic core and a non-magnetic coating. The core consists of nanometre sized, monodispersed iron oxide particles of magnetite (Fe^3^O^4^) and/or maghemite (γ-Fe^2^O^3^). These are organized in a crystalline pattern, which is controlled by the synthesis procedure just as the core’s composition [17, 18].

Since bare iron oxide particles are insoluble in water, tend to agglomerate and precipitate, an appropriate coating by a polymer shell is crucial. The coating stabilizes the nanoparticles while its chemistry also determines the fate of the compound since it is the polymer shell, the host body first interacts with [18]. Throughout the literature substances such as carboxylates, phosphates, silica, dextrans, polyethylene glycol (PEG) and chitosan have been proposed for coating [19–24]. However, most FDA-approved ION-based contrast agents are coated with polysaccharide dextran or its derivatives (Table 1). Despite further chemical aspects, dextrans are favourable for their intrinsic affinity to iron oxide particles. In addition, non-toxicity and good physiological tolerance have been proven in other applications of dextrans [25].

**Table 1:** Representative examples for iron oxide-containing contrast agents

Coating	Examples	Size in nm	Comment
Carboxymethyl- dextran	Ferumoxytol (Feraheme™)	17-31	FDA approved for anaemia and in clinical use
Carboxydextran	Ferucarbotran (SHU 555A, Resovist®)	45-60	FDA approved. Discontinued from clinical use in 2009.
Dextran	Ferumoxtran (USPIO, AMI-227, NC100150, BMS-180549)	17-21	Discontinued after 37 clinical studies phase I-III in 2010.
Dextran	Ferumoxides (Feridex™)	100	Discontinued from clinical use in 2008.
Crosslinked dextrans	CLIO-Cy5.5	30-35	Experimental use
Citrate	VSOP-C184	4-7	Experimental use, clinical phase I study in 2004

Representative examples for iron oxide-containing contrast agents for CMR with clinical, commercial and/or considerable experimental use adopted and modified from Weissleder et al. [34]. Additional information and further iron oxide-based contrast agents can be seen elsewhere [40]. CLIO: cross-linked dextran iron oxide nanoparticles

### Synthesis of iron oxide nanoparticles

Numerous synthesis pathways focusing on good reproducibility and the desired combination of core and coating have been examined. According to Laurent et al. the simplest and most efficient protocols involve co-precipitation techniques of ferrous (Fe^2+^) and ferric (Fe^3+^) salts in an aqueous medium with the pH adjusted [18, 26]. The formulation of the final composition is achieved by applying a stabilizing base, which is actively steered for coating [18, 27]. Techniques that are more sophisticated include e.g. microemulsions, hydrolysis and thermolysis of precursors or flow-injection methods as described elsewhere [28–30]. However, throughout well-known procedures, the synthesis of uniform sized particles remains challenging. Thus, IONs rather show a narrow distribution in diameter than distinct measures [18]. Moreover, the total particle size is additionally enlarged in solution due to solvation [12]. According to this hydrodynamic diameter, IONs can be class-divided as follows: Micrometre-sized iron oxide particles are named MIONs; IONs measuring 50 nm to 250 nm are referred to as SPIO (superparamagnetic iron oxide particles), while those ranging from 20 nm to 50 nm in diameter are named ultra-small SPIO (UPSIO) and even smaller IONs (<20 nm) are declared as very-small SPIO (VSPIO) [31].

## Major properties of the core and polymer shell of iron oxide nanoparticles

### Superparamagnetism

Iron oxide nanoparticles are superparamagnetic and can be magnetized in CMR. Superparamagnetic materials consist of ferri- or ferromagnetic nanoparticles that can only build up a single magnetic domain. For small particles such as IONs, thermal energy is sufficient to randomly flip their magnetic orientation [12]. Thus, in the absence of an externally applied field their net magnetization appears to be in average zero. However, in the presence of a magnetic field their magnetic moments align, which leads to a much larger magnetic susceptibility than those observed for paramagnetic agents [32]. This phenomenon is physically described by Neel- and Brown-relaxation [33].

In the context of ceCMR, superparamagnetic IONs are referred to as negative contrast agents. Their induced magnetic field alters the relaxation properties in neighbouring tissue, resulting in a signal void in T2/T2*-weighted images. Thus, the performance of an ION-based contrast agent can be quantified by the decrease in transverse relaxation times or the increase in relaxation rate R2 (R2 = 1/T2). In general, when comparing the same particle doses, the effect of contrast enhancement is higher for IONs than for gadolinium-based contrast agents [12].

### Uptake & blood circulation time

The main factors determining pharmacokinetics and organ distribution of IONs are the biophysical characteristics and their surface chemistry such as composition, density and charge [34]. Depending on these factors, most IONs are internalized by cells of the RES and accumulate in the liver, spleen and kidneys following intravenous administration. SPIOs tend to accumulate in the spleen while particles sized up to micrometres are preferentially filtered by the liver [27, 34]. In contrast, due to their smaller size, USPIOs show a longer blood circulation time and are removed from the blood pool through extravasation and renal clearance.

Cellular uptake of IONs is primarily associated with phagocytosis. According to Weissleder et al. most dextran-coated IONs are phagocytosed by circulating monocytes or tissue resident macrophages [34]. Due to their large surface-to-volume ratio, IONs tend to adsorb plasma proteins such as opsonins which subsequently induce phagocytosis [35]. Particularly IONs having a surface charge that can induce bonds to phagocytes through electrostatic interaction show increased adsorption [36]. However, cellular uptake is not only limited to phagocytosis and monocytes or macrophages [34, 37]. Amongst others, dendritic, cancer and endothelial cells also take up IONs. Further research is needed to examine the kinetics of ION uptake by different monocyte and macrophage subsets as well as other cell types.

Internalization of IONs results in a decreased blood circulation time, limiting the likelihood to reach the desired/targeted tissue or organ. Several strategies to prolong their blood half-life or to hide them towards cells of the RES have been discussed in the literature. In principle, particles possessing the following properties have shown a relatively increased blood circulation time: (i) hydrophilic coatings, (ii) hydrodynamic diameters smaller than 50 nm, (iii) neutral or near neutral charged surface designs and (iv) shielding groups added to the surface such as PEG that block electrostatic or hydrophobic interactions between opsonins and particle surface [19, 25, 38].

### Targeting & drug carrier

Apart from imaging purposes, IONs can also be used as targeted carriers for drugs or small molecules. Due to their large surface-to-volume ratio, IONs enable the integration of multiple functional groups using appropriate surface chemistry [16]. As reviewed by Sun et al. various studies report on the successful conjugation of targeting agents, permeation enhancers, optical dyes or therapeutic agents on the surface of IONs [32]. Linking between surface and functional groups was achieved by electrostatic adsorption and (amongst others) by covalent or oxidative conjugation strategies. Appropriate bindings must be stable in aqueous ionic solutions at physiological pH, while ligands must not interfere with non-targeted tissues and should not decrease blood circulation time or introduce toxicological effects [18]. Again, many strategies have been reported aiming at minimizing detrimental effects, e.g. cross-linking IONs via their coating material as introduced by Wunderbaldinger and colleagues [39]. They were able to bind dextran coated IONs by epichlorohydrin and ammonia, forming complexes classified as CLIO (cross-linked iron oxide). These compounds exhibit a flexible platform for functionalization – but did not reach clinical approval yet [40].

Besides active targeting via functional groups, IONs can also accumulate at targeted organs/tissues by the help of an external magnetic field [41–43]. Exploiting the ION’s large magnetic moment, a sufficiently high magnetic gradient can exert drag forces from blood flow. Such an approach allows an enhanced retention and/or accumulation of IONs at the target site [44]. This approach offers great potential for applications combining molecular imaging and controlled drug release – sometimes referred to as theranostics [17, 44]. In comparison to non-navigated systemic drug application, externally navigated ION-complexes may theoretically lead to a decrease in the required dose and in adverse side effects in non-targeted organs while enabling monitoring of therapy at the same time.

### Safety and toxicity

Mechanisms leading to cellular damage or impairment of cellular viability are primarily related to surface chemistry and functionalization of the IONs. In this context, cellular uptake and subsequent degradation of the IONs have to be considered. According to Singh et al. cell exposure to IONs could theoretically induce toxic side effects such as impaired mitochondrial function, activation of inflammatory or apoptotic pathways with formation of apoptotic bodies, generation of reactive oxygen species and even DNA damage [36, 45]. However, in vitro studies comparing different metal oxide nanoparticles convincingly showed that ION concentrations below 100 μg/ml are safe and non-cytotoxic [46]. Moreover, human in vivo studies examining the safety of ferumoxtran-10 or ferumoxytol reported a convincing safety profile with only rare occurrence of mostly mild and short lasting side effects (such as urticaria, diarrhoea or nausea after application) – if the respective suggestions regarding the administration are appropriately considered [47, 48]. It is assumed that these rare side effects are mainly associated with the endogenous degradation pathway of iron [45, 47, 49]. However, although serious side effects are exceedingly rare with USPIO, there have been reports of fatal hypersensitivity responses to ferumoxytol.

## Pre-clinical applications of iron oxide nanoparticles

### Myocardial infarction model

A major goal of performing ceCMR after myocardial infarction (MI) is to differentiate injured from (remote) healthy myocardium. In this context, Chapon et al. evaluated dextran-coated USPIOs in a rodent model of permanent ligation of the left coronary artery (LCA). Concentrations of 5 and 10 mg Fe/kg were administered once after 5 h, 24 h and 48 h of occlusion followed by T2-weighted MR acquisitions. Subsequent image analysis revealed higher contrast between healthy and ischemic myocardium in series acquired at later stages. Furthermore, these authors identified a negative linear relationship between contrast enhancement and the elapsed time since induction of MI. However, doubling the concentrations of IONs did not significantly increase the observed contrast [50].

Since macrophages are primarily involved in the regulation of post-MI wound healing, Sosnovik and colleagues studied the infiltration of macrophages in the infarcted tissue. Permanent ligation of the LCA was performed in mice followed by the administration of varying concentrations of a magnetofluorescent USPIO. Forty-eight hours after ION injection, T2-weighted CMR of the myocardium showed a negative contrast enhancement in the left ventricular (LV) anterior and adjacent walls – that was not observed in sham mice. Calculated contrast-to-noise-ratio (CNR) between remote (healthy) myocardium and injured LV wall segments was significantly higher for mice treated with USPIOs. Moreover, average CNR increased with increasing ION concentration. Since these findings were confirmed by ex vivo immunohistochemistry (proving co-localization of USPIO and macrophages), the authors concluded that visualization of labelled macrophages is feasible in vivo and allows the assessment of myocardial injuries after infarction [51].

In contrast to the aforementioned studies, Montet-Abou et al. investigated labelling of resting monocytes and macrophages prior to occlusion-reperfusion of the left anterior descending artery (LAD). Labelling was aimed at with polymer-coated VSPIOs that were administered to rats three days prior to LAD occlusion. This duration was chosen in order to ensure total clearance of VSPIOs from the blood pool. Following reperfusion, T2-weighted CMR was repeated every 24 h. In doing so, hypointense areas indicative of infarcted myocardium were only visible in rats treated with IONs. Additional mapping of T2-values revealed an increase in signal void (negative enhancement) over elapsed time since reperfusion. This temporal signal change was consistent with an increasing number of pre-loaded monocytes and macrophages found in infarcted tissues by histology. Additional MR-tagging studies showed a relationship between hypointense areas and hypokinetic segments. In summary, the authors could demonstrate that pre-loading and subsequent tracking of ION-labelled macrophages is suitable to depict changes in infarct-associated inflammatory diseases [52].

**Fig. 3:**
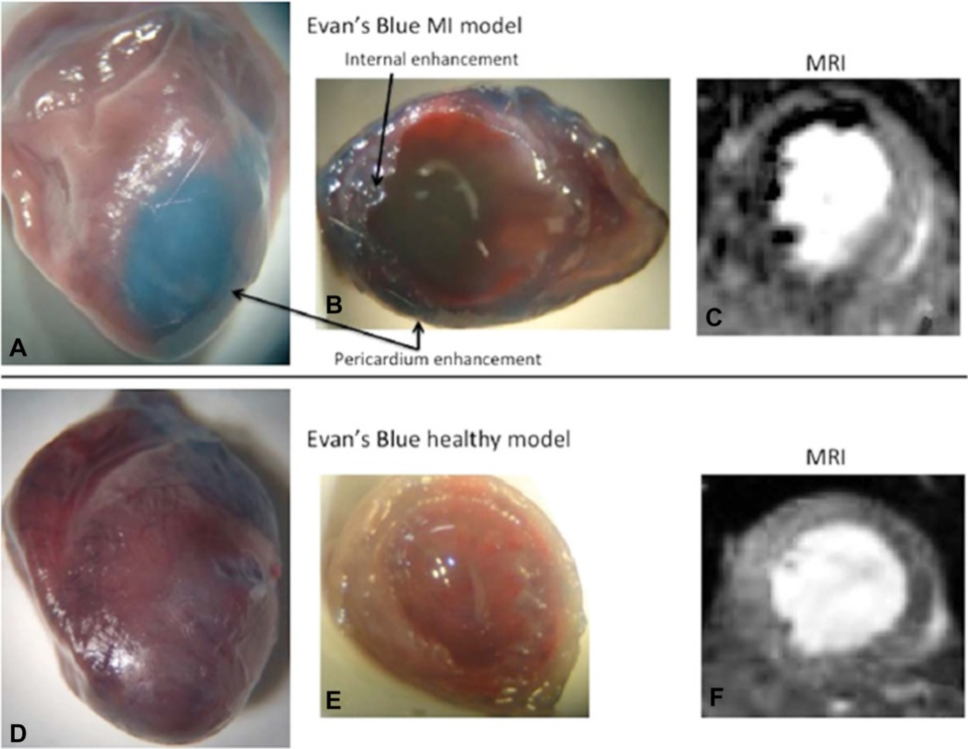
Comparison of explanted hearts from mice after induced myocardial infarction (top row) and after sham procedure (bottom row). Images **a** and **d** show myocardium stained with Evans blue indicating areas with enhanced permeability in the area of myocardial infarction (**a**, arrows). The corresponding sections display damaged tissue only for infarcted hearts (**b** vs. **e**) and corresponding signal void in T2-weighted MR images after infusion of VSPIO (**c** vs. **f**). Reproduced with permission from Protti et al. [53]

In a recent study, Protti et al. extensively investigated the performance of VSPIOs in a murine model of ischemia-reperfusion [53]. The IONs were administered according to four different experimental setups: one pre-MI protocol that was based on the application of VSPIOs seven days before surgery, and three post-MI protocols that differed in the delay between reperfusion and injection (24 h, 6d, 28d). The first post-MI protocol included MR imaging and T2*-mapping 2 h, 48 h and 7d after VSPIO application while mice in the other setups were imaged solely after 48 h. Image analysis of the pre-MI protocol showed no negative contrast enhancement, but a decrease in functional parameters. While the latter was related to myocardial injury, the absence of a negative contrast enhancement was explained by reperfusion of the infarcted territory. On the contrary, CMR series of the first post-MI protocol showed signal voids associated to VSPIO accumulation (Fig. 3). Moreover, Protti and colleagues found a temporal correlation between the recovery of T2*-values and the decrease of areas with ION-induced signal extinction. This correlation was not consistent with functional and volumetric measurements and did not apply for MR imaging six days post-MI as performed in the second setup. Furthermore, no signal void was seen on CMR scans 28 days after MI indicating the restoration of vascular integrity and/or the endpoint of remodelling. Histological examinations performed 48 h post-injection showed a high iron density at the inner endocardial borders of infarcted areas. In contrast, no or little iron was identified in epicardial borders. However, macrophages were identified on both sites. Consequently, the number of macrophages did not significantly correlate with T2*-values. Hence, the authors argued that other cells apart from monocytes and macrophages internalized VSPIOs. Additional electron microscopy showed an augmented VSPIO compartmentalization in macrophages located in endocardial areas as opposed to epicardial regions. Protti and colleagues assumed that an enhanced VSPIO-leakage into endocardial tissue might explain their findings. Increased leakage in turn might have originated from locally increased vessel permeability along with a decreased tissue perfusion [53].

### Myocarditis model

To the best of our knowledge, there is only one pre-clinical animal study published so far that addresses ION-administration and CMR of inflammatory processes associated with myocarditis: In 2012, Moon and colleagues presented the results of their comprehensive rodent study, examining the feasibility to detect and discriminate different grades of experimental autoimmune myocarditis (EAM) by the help of SPIOs. They used IONs covered by a fluorophore-incorporated silica coating that were additionally conjugated with PEG polymer chains. Experimental setups involved injection of 10 mg Fe/kg on day 21 after induction of EAM or 24 h before CMR, respectively. Histological examinations comprising different staining and microscopy techniques were performed after imaging [54]. First, ex vivo imaging of inflammatory cellular infiltrates was performed in rats at day 21 after induction of EAM and 24 h after SPIO-injection. Markedly enlarged hearts with dilated ventricles, thickened myocardium and scattered inflammatory foci of varying sizes were observed. Moreover, histological analysis revealed a dense accumulation of macrophages in pathologically altered areas with SPIOs compartmentalized in lysosomes [54].

To evaluate in vivo assessment of EAM using SPIO-based ceCMR, Moon and collaborators determined the CNR of the LV septum between pre- and post-SPIO images. T2-weighted images acquired 30 min after injection of SPIOs showed homogenous negative contrast enhancement in ventricle walls of both EAM and control rats indicating SPIO accumulation. Consequently, CNR was significantly decreased. While contrast normalized after 24 h in the control group, areas of negative contrast enhancement persisted in EAM rats. In accordance to histological findings, the authors assumed that IONs were present in the vasculature after the injection in both groups, but were washed-out from site afterwards in control rats. Further, they presume that inflammatory cells including macrophages infiltrated affected tissue and phagocytosed SPIOs preventing their washout in rodents infected with EAM [54].

To evaluate the diagnostic yield of SPIO-based ceCMR regarding discrimination of histologically defined grades of myocardial inflammation, CMR was performed at different days after induction of EAM. A linear relationship was found between changes in CNR and stained areas of both inflammation and macrophage counts for all grades. However, the authors did not observe a significant difference in CNR between moderate and severe forms of EAM [54].

**Fig. 4:**
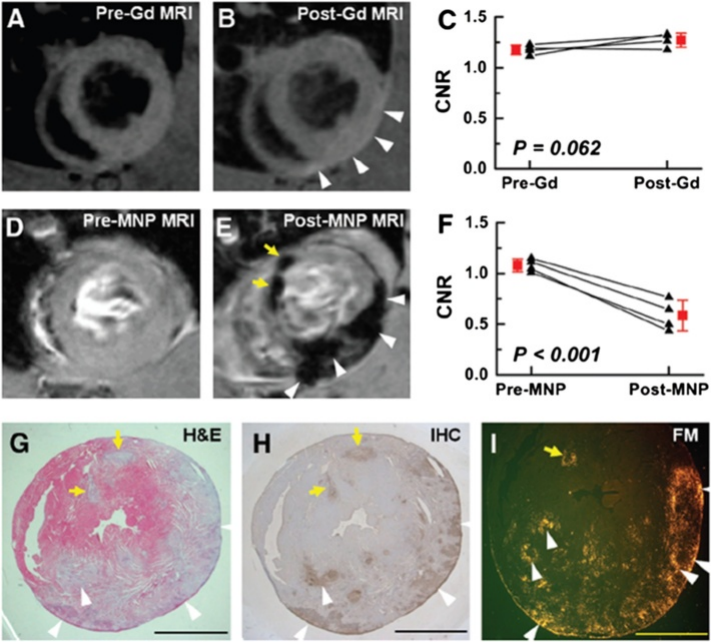
Comparison between SPIO-CMR and LGE-CMR. T1-weighted LGE-CMR before (**a**) and after (**b**) gadolinium injection, and T2*-weighted SPIO-CMR before (**d**) and after (**e**) SPIO injection in the same EAM rats are compared. Whereas weakly enhanced areas (**b**, white arrowheads) are visualized in the left ventricular wall in the LGE-CMR, the positively enhanced area becomes negative contrasted (**e**, white arrowheads) in SPIO-CMR. Magneto-fluorescent nanoparticle CMR provides more specific identification of inflammatory lesions (**e**, yellow arrows) than LGE-CMR and more pronounced changes in CNR (**c** and **f**). The negative contrast regions in the SPIO-CMR are in good agreement with inflamed areas in H&E images (**g**), macrophage infiltrations in IHC images (**h**), and SPIO-fluorescence spots in FM (**i**). CNR: contrast-to-noise ratio; H&E: haematoxylin and eosin; IHC: immunohistochemistry; FM: fluorescence microscopy. Reproduced with permission from Moon et al. [54]

In another experimental setup, the distinctiveness of SPIO-based ceCMR was compared to T2-weighted images as well as to early (EGE) and late-gadolinium-enhanced (LGE) CMR. All images were acquired sequentially including SPIO-based ceCMR in the same rats. In doing so, gadolinium-based ceCMR showed large areas of inflammation, but no small foci that were visible in SPIO-based ceCMR (Fig. 4). Since these rather small inflammatory areas were confirmed by staining, the authors deduced a superior diagnostic accuracy for IONs. This observation is in accordance with the patchy pattern of necrosis and viable myocytes in myocarditis that makes appropriate detection by a gadolinium-based contrast-approach challenging [54].

Additional studies addressed the issue of assessing the evolution/disease course of EAM. IONs were administered in the same rats at day 15 and 20, respectively, after the induction of EAM. At day 16, SPIO-based ceCMR showed several inflammatory spots with significantly decreased pre- vs. post-SPIO CNR. In comparison, different contrast patterns and areas that tended to have an even lower CNR were observed at day 21. Furthermore, repeated CMR studies of EAM-rats 24 h, 72 h and 120 h after SPIO application at day 18 showed no migration of labelled macrophages into the neighbouring myocardium [54].

## Clinical applications of iron oxide nanoparticles for myocardial inflammation imaging

Although many preclinical studies in different animal models and with different IONs rather consistently demonstrated that non-invasive visualization of myocardial inflammation with CMR is possible and based on in vivo and/or ex vivo labelling of monocytes/macrophages (Table 2), the number of clinical studies aiming at ION-based depiction of myocardial inflammation is quite limited. So far, clinical studies with IONs were only performed in patients with acute myocardial infarction and no clinical data are available in patients with myocarditis or other inflammatory diseases of the heart that are not caused by coronary artery disease.

**Table 2:** Overview of cited pre-clinical animal studies

Object of study		Field-strength B0 in T	# Animals	Dose in mg Fe/kg	Average particle size in nm	Coating	Half-life in h*	Relaxivites in mM^−1^ s^−1^
Myocardial infarct	Chapon et al. 2003 [50],	7.0	42	1, 5, 10	50	Dextran	0.8	R1 = 1.2, R2 = 24; (7 T)
	Sosnovik et al. 2007 [51],	9.4	21	3, 10, 20	32	Cross-linked dextrans	10	
	Montet-Abou et al. 2009 [52],	1.5	44	10	15	Polymer	6	
	Protti et al. 2014 [53],	7.0	73	15	7	Citrate	2	R1 = 30, R2 = 39; (0,47 T)
Myocarditis	Moon et al. 2012 [54],	4.7	90	10	85	Silica	2.8	R2 = 130; (4,7 T)

Overview of cited pre-clinical animal studies, including particle doses used and physical properties - partially adapted from MICAD [40]. * half-life in rodent blood

The first clinical trial in patients with acute myocardial infarction (entitled NIMINI-1) was performed by our group using ferucarbotran, a SPIO approved at that time as Resovist® for a non-cardiac indication [55]. Twenty patients with either an acute ST-elevation MI (STEMI) or non-ST-elevation MI (NSTEMI) were included to this pilot-study. After coronary angiography, a first baseline CMR study was performed within seven days after onset of cardiac symptoms and a second CMR study at different time points (10 min to 48 h) after intravenous single-dose injection of ferucarbotran. Following ferucarbotran administration, the area of myocardial hypoenhancement in T2-/T2*-weighted images (suggestive of either SPIO accumulation and/or intramyocardial haemorrhage) did not exceed the area of conventional LGE and no statistical significant difference were seen when pre-SPIO images were compared to post-SPIO ones. Hence, T2-/T2*-weighted CMR imaging using the approved dose of ferucarbotran (39 mg Fe/patient) did not result in an improved visualisation of inflamed/infarcted myocardium compared to conventional gadolinium-based necrosis/fibrosis imaging in this trial.

However, the reasons for this disappointing result were quite obvious: There is a linear relationship between the applied dose of U/SPIO and the degree of induced hypoenhancement in T2-/T2*-weighted images. The minimal concentration successfully used in animal models was 2-3 mg Fe/kg. Unfortunately, the approved dose of Resovist® (for patients with suspected liver disease) was one single injection of 1.4 ml Resovist® - corresponding to a dose of 39 mg Fe per patient. Due to medicolegal reasons, our group was not allowed to use a different dose at that time. Thus, the maximal Fe concentration after administration of Resovist® (e.g. in a 60 kg patient) was only 0.65 mg Fe/kg. Moreover, Resovist®’s blood half-life was possibly too short (<15 min) for efficient uptake in macrophages and accumulation in the infarcted myocardium.

Recently, the group of Newby and co-workers from Edinburgh and our group independently (and at the same time) performed new clinical trials in patients with acute STEMI using for the first time ferumoxytol, an USPIO that was approved by the FDA for treatment of anaemia in patients with renal disease. The group from Edinburgh studied 10 patients (and 6 controls) and used a ferumoxytol concentration of 4 mg Fe/kg. CMR scans comprising T2*-weighted sequences were performed on a 3-T scanner within five days of admission at baseline, 24 h and 48 h and R2* values were calculated for specific regions of interest. In regions with infarcted myocardium, the R2* value increased from 41.0 ± 12.0 s(−1) at baseline to 155 ± 45.0 s(−1) (p < 0.001) at 24 h and to 124 ± 35.0 s(−1) (p < 0.05) at 48 h. Interestingly, a similar, however, lower change in R2* was documented in the remote (non-infarcted) myocardium while no change at all was detected in the skeletal muscle in this study [56].

**Fig. 5:**
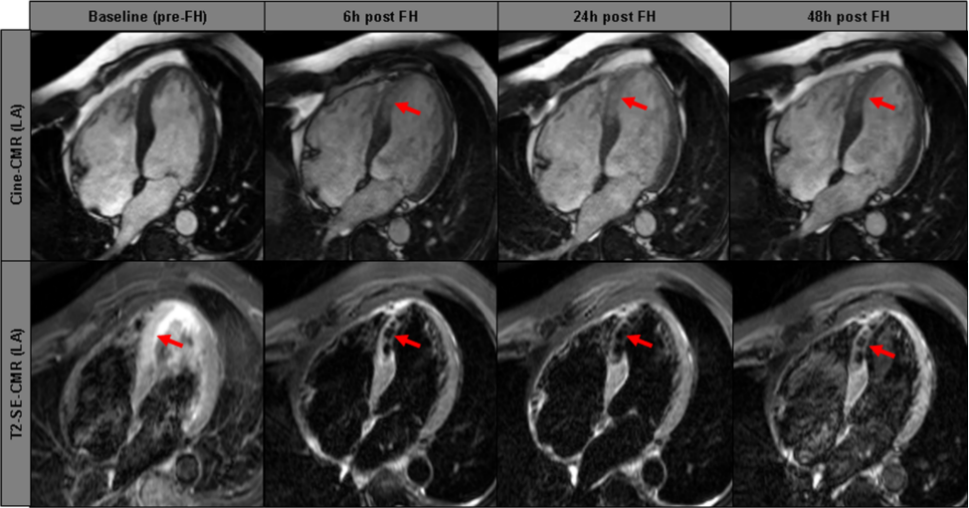
Exemplary cardiovascular magnetic resonance images in the long-axis view of a study patient with a septal myocardial infarction who underwent pre-Feraheme^™^ (FH) (baseline) and post-Feraheme^™^ (after 6, 24, 48 h) cardiovascular magnetic resonance imaging studies, respectively. The first line shows cine-cardiovascular magnetic resonance images at different time points with proof of hyperenhancement in the septal wall at 6–48 h post-Feraheme^™^ (red arrows). The second line shows T2-weighted short-tau inversion recovery-spin echo images at different time points with proof of hypoenhancement in the septal wall at 6–48 h post-Feraheme^™^ (red arrows). Reproduced with permission from Yilmaz et al. [57]

Our clinical phase-III trial with ferumoxytol (entitled NIMINI-2) comprised 14 patients with acute STEMI. After a first baseline CMR study (pre-USPIO) on a 1.5-T scanner, a follow-up CMR study (post-USPIO) was performed 48 h after administration of ferumoxytol. In this trial, the approved dose of ferumoxytol (containing 510 mg Fe in 17 ml solution) was used - resulting in a concentration of e.g. 8.5 mg Fe/kg in a 60 kg subject - while the median dose of iron was 5.4 mg Fe/kg in the NIMINI-2 study. The median extent of T2-weighted hypoenhancement in the region of myocardial infarction - which was not present in baseline studies in any patient (!) - was 19% (IQR 14-22%) (Fig. 5). Similar to the aforementioned study, a substantial drop in absolute T2*-values (corresponding to an increase in R2*-values) was observed not only in the infarct core and peri-infarct zone, but also in the remote (healthy) myocardium. In addition, there was only a minor change in the T2*-value of the skeletal muscle. Additional ex vivo analyses showed that a substantial ferumoxytol uptake was detected only in cultured macrophages, but not in peripheral blood monocytes from study patients. Taken together, we could demonstrate in humans that an USPIO-based approach allowed detailed characterization of infarct pathology by causing hypoenhancement (in T2-weighted images) and signal void (in T2*-mapping images), mainly by detecting infiltrating macrophages and most likely by altered tissue partitioning of USPIOs in different layers of the infarcted myocardium [57].

Compared to ferucarbotran (an SPIO that was used in the NIMINI-1 study), ferumoxytol is an USPIO with a mean diameter of only ~30 nm, a neutral surface charge, a magnetic relaxivity of r1 = 15 mmol/L/s and r2 = 89 mmol/L/s (at 1.5 T) and a blood half-life of ~15 h. Such a long blood half-life that is due to its small size and neutral surface charge is a prerequisite for accumulation of such particles (during continuous blood circulation) in the inflamed/infarcted myocardium. Although ferumoxytol is not approved as an CMR contrast agent so far, the specific magnetic relaxivity properties of ferumoxytol do allow both T1-weighted as well as T2-/T2*-weighted MR imaging – as was shown in the NIMINI-2 study. Noteworthy, ferumoxytol was developed by AMAG Pharmaceuticals, Inc., in the USA and was first approved as Feraheme^TM^ for iron replacement therapy in patients with anaemia due to chronic renal failure in June 2009 by the FDA. Subsequently, in June 2012 the company Takeda received European marketing authorization for Rienso® (i.e. 510 mg ferumoxytol) and started to launch Rienso® across Europe. Unfortunately, Rienso® is no longer marketed in Europe due to commercial reasons. Moreover, since ferumoxytol is not renally eliminated, it can also be safely administered to patients with severe renal dysfunction – in contrast to gadolinium-based compounds.

## Current challenges and future promises of iron oxide nanoparticles for myocardial inflammation imaging

From a clinical point-of-view, the opportunity to image/visualize infiltrating macrophages in the inflamed myocardium will be of potential diagnostic as well as therapeutic value since the extent and degree of myocardial disease might be monitored more accurately and therapeutic success might be assessed more appropriately. Moreover, USPIOs such as ferumoxytol may help to differentiate acute myocardial inflammation from rather chronic myocardial disease (such as myocardial fibrosis) – which is not possible with current conventional gadolinium-based approaches. In this context, future applications of USPIO may also comprise other cardiac diseases that are characterised by myocardial inflammation such as cardiac transplant rejection or cardiac sarcoidosis.

Recently, two distinct phases in the process of infarct healing were described [58]: a first inflammatory phase (until day ~4 following acute MI) that is characterized by the accumulation of pro-inflammatory monocytes (CD16^−^) that primarily remove necrotic cells and debris, and a second resolution phase (following day ~4 after acute MI) in which reparative monocytes (CD16^+^) predominate and orchestrate myocardial tissue repair. In addition, recent preclinical studies suggest that a misbalance between these two phases (e.g. an excessive accumulation of inflammatory monocytes during the first phase) may have adverse effects on infarct healing [59]. Therefore, future imaging studies need to consider such complex and sophisticated mechanisms and aim at detecting specific subsets of monocytes/macrophages in the human myocardium in order to allow an improved understanding of monocyte trafficking in the inflamed heart. Obviously, USPIO-based imaging of monocytes/macrophages that was performed in clinical studies so far did not (and still do not) allow such a subtle differentiation of monocyte/macrophage subsets.

Moreover, it has to be expected that monocyte/macrophage trafficking in case of non-ischemic inflammatory diseases such as myocarditis will show different patterns of monocyte/macrophage activation with different cell subsets being involved. Hence, the affinity of IONs to monocytes/macrophages involved in acute (human) myocarditis might be different compared to those ones orchestrating inflammation in case of myocardial infarction. Future clinical studies with comprehensive myocardial tissue as well as blood analyses will hopefully allow evaluating such sophisticated differences.

Although (passive) uptake of IONs by infiltrating monocytes/macrophages seems to be the major mechanism leading to myocardial signal void on T2-/T2*-weighted CMR images, the pre-clinical data of Protti et al. [53] as well as the clinical data from the NIMINI-2 trial [57] suggest that altered tissue partitioning of USPIOs in different layers of the infarcted myocardium – independent of the amount of macrophages (!) - needs to be considered carefully. Whether the degree of microvascular obstruction and/or the presence and frequency of other cell types (apart from monocytes and macrophages) constitutes another reason for this observation needs to be evaluated in future studies.

Finally, the versatile coating properties of USPIOs in addition to the possibility to navigate such particles to the targeted organ/tissue by using external permanent magnets, make these particles highly suitable as versatile drug-carriers for targeted therapy. Since stem cell transplantation is a promising strategy for therapy of acute myocardial infarction and of CAD-driven heart failure, USPIO may also be used to label, target and subsequently visualize such cells in vivo using CMR. Recently, Cheng et al. [60] performed a very elegant preclinical study and applied targeted nanomedicine to achieve the following effects at the same time: a) in vivo cell-mediated repair of infarcted myocardial tissue, b) non-invasive CMR-based therapy monitoring using USPIO and c) targeted accumulation of bone marrow-derived stem cells in the infarcted myocardium, however, without cellular transplantation. These goals were achieved by linking USPIO with two types of antibodies: An anti-CD45 antibody was used to target exogenous bone marrow-derived stem cells (expressing CD45) and an anti-MLC antibody to target injured cardiomyocytes (expressing MLC). After infusion of such USPIO (linked with two different antibodies), accumulation of bone marrow-derived stem cells in the infarcted area was achieved by an external magnet (1.3-T) that was positioned above the left thoracic region during USPIO infusion and continuously for 10 min after infusion. These authors’ results indicate that scar formation may be reduced and left ventricular function improved by using such an elegant approach. Hence, IONs such as USPIO could have far-reaching consequences for future developments regarding molecular diagnostics as well as targeted therapy.
